# A gp41 MPER-specific llama VHH requires a hydrophobic CDR3 determinant for neutralization but not for antigen recognition

**DOI:** 10.1186/1742-4690-9-S2-P317

**Published:** 2012-09-13

**Authors:** W Weissenhorn, D Lutje Hulsik, M Hock, YY Liu, NM Strokappe, ME Khattabi, JP Langedijk, LE McCoy, A Forsman-Quigley, MM Aasa-Chapman, RA Weiss, TC Verrips, L Rutten

**Affiliations:** 1 Grenoble University, Grenoble, France; 2 Biomolecular Imaging (BMI), Faculty of Science, Utrecht University, Utrecht, the Netherlands; 3 Pepscan Systems BV, Lelystad, the Netherlands; 4 MRC Centre for Medical Molecular Virology, University College London, London, UK

## Background

The membrane proximal external region (MPER) of the HIV-1 glycoprotein gp41 is targeted by broadly neutralizing antibodies such as 2F5, 4E10 and Z13, which recognize antigen and membrane components.

## Methods

In this study, we immunized llamas with gp41 proteoliposomes and selected a MPER-specific single chain antibody (VHH), 2H10, whose gp41 epitope overlaps with that of mAb 2F5.

## Results

2H10 binds to the intermediate conformation of gp41 with medium nanomolar affinity. Construction of 2H10 biheads (bi-2H10) increases the binding affinity by a factor of 20. Bi-2H10 neutralizes various sensitive and resistant HIV-1 strains, as well as SHIV strains in a TZM-bl cell assay. We further present structural data from crystallographic and NMR analyses together with mutagenesis data that allowed to map the interaction site on gp41. This revealed that 2H10 has a long CDR3 whose tip exposes a tryptophan residue that is not required for gp41 interaction, but crucial for neutralization.

## Conclusion

Our data indicate that 2H10 induced by immunization classifies as a functional MPER antibody as a bihead that requires both antigen recognition and membrane interaction for neutralization.

